# Complete and Assembled Genome Sequences of Pantoea calida DSM 22759^T^ and Pantoea gaviniae DSM 22758^T^

**DOI:** 10.1128/genomeA.00157-18

**Published:** 2018-03-22

**Authors:** Marc J. A. Stevens, Katrin Zurfluh, Roger Stephan

**Affiliations:** a Institute for Food Safety and Hygiene, Vetsuisse Faculty, University of Zurich, Zurich, Switzerland

## Abstract

The genomes of Pantoea calida DSM 22759^T^ and Pantoea gaviniae DSM 22758^T^ were sequenced using single-molecule real-time sequencing. They consist of a 4.3-Mbp chromosome containing 4,092 genes, of which 3,977 encode proteins, and a 4.5-Mbp chromosome containing 4,236 genes, of which 4,120 encode proteins, respectively.

## GENOME ANNOUNCEMENT

The genus Pantoea belongs to the order of Enterobacteriales and is, since the taxonomic update of the family Enterobacteriaceae, placed in the family Erwiniaceae (1). The two species Pantoea gaviniae and Pantoea calida form a distinct clade within the genus Pantoea and are closely related (average nucleotide identity, 88%) but have an average nucleotide identity of only 73.44 to 78.48% with other members of the genus (2). Here, the completely sequenced and assembled genomes of the type strains Pantoea calida DSM 22759 and Pantoea gaviniae DSM 22758, originally isolated from infant formula and an infant formula production environment, are presented.

DNA was isolated using the Wizard genomic DNA purification kit (Promega AG, Dübendorf, Switzerland). Sequencing was performed using single-molecule real-time sequencing on 2 PacBio RS II cells (Pacific Biosciences, Menlo Park, CA, USA) at the Functional Genomics Center Zurich, Switzerland. Raw reads were filtered using the RS filter only protocol in the SMRT Portal (Pacific Biosciences) with 0.85 as the polymerase read quality cutoff and a minimal length of 500 bp. For Pantoea calida DSM 22759^T^, a total of 81,593 reads with an average length of 15,916 bp passed filtering, corresponding to 1,298,688,643 sequenced base pairs and a coverage of approximately 300-fold. The reads were assembled using the Canu assembler (3) with the option “pacbio-raw” and an estimated genome size of 4.4 Mbp. The Canu output consisted of two contigs, the first containing the chromosomal sequence and the second a phage sequence, as identified using PHAST (4). The phage sequence was not submitted to GenBank. The chromosomal contig was polished in CLC Workbench 7 (CLC, Aarhus, Denmark) and is 4,308,453 bp long. The G+C content of the chromosome is 57.0%. The start of the chromosome was identified using Ori-Finder (5), and the genome was annotated using the NCBI Prokaryotic Genomes Annotation Pipeline server. The genome of Pantoea calida DSM 22759^T^ contains 4,092 genes, of which 3,977 genes encode proteins. Further, 83 tRNA genes and 7 rRNA operons were found to be present.

For Pantoea gaviniae DSM 22758^T^, a total of 60,962 reads with an average length of 10,288 bp were selected, resulting in 627,151,760 sequenced base pairs and a coverage of approximately 140-fold. The reads were assembled using the Canu assembler 1.6 (3) with the option “pacbio-raw” to an estimated genome size of 4.5 Mbp. The Canu output consisted of a single contig that was subsequently polished in CLC Workbench 7 (CLC, Aarhus, Denmark). The chromosomal origin of replication was identified using Ori-Finder 5.0 (5) and the start of the genome set at 9 bp upstream of the first DnaA box in the identified origin region. The genome of Pantoea gaviniae DSM 22758^T^ consists of a circular 4,527,605-bp chromosome with a G+C content of 58.0%. The genome was annotated using the NCBI Prokaryotic Genomes Annotation Pipeline server. The chromosome contains 4,236 genes, of which 4,120 encode proteins. Further, 7 rRNA operons and 86 tRNA genes were identified.

### Accession number(s).

The sequence and annotation data of the complete genomes of Pantoea calida DSM 22759^T^ and Pantoea gaviniae DSM 22758^T^ are deposited in the GenBank database with the accession numbers CP026378 and CP026377, respectively.
